# Classification of Severity of Lung Parenchyma Using Saliency and Discrete Cosine Transform Energy in Computed Tomography of Patients With COVID-19

**DOI:** 10.1155/ijta/4420410

**Published:** 2025-01-06

**Authors:** Santiago Tello-Mijares, Francisco Flores, Fomuy Woo

**Affiliations:** ^1^Basic Sciences Department, Instituto Tecnológico de la Laguna Tecnológico Nacional de México Campus Laguna, Torreón, Coahuila, Mexico; ^2^Postgraduate Department, Instituto Tecnológico de la Laguna Tecnológico Nacional de México Campus Laguna, Torreón, Coahuila, Mexico; ^3^Medical Familiar Unit, Instituto de Seguridad y Servicios Sociales de Los Trabajadores del Estado, Torreón, Coahuila, Mexico

**Keywords:** artificial intelligence, computed tomography, COVID-19, medical diagnostic imaging, SARS-CoV-2 virus

## Abstract

This study proposes an automated system for assessing lung damage severity in coronavirus disease 2019 (COVID-19) patients using computed tomography (CT) images. These preprocessed CT images identify the extent of pulmonary parenchyma (PP) and ground-glass opacity and pulmonary infiltrates (GGO-PIs). Two types of images—saliency (*Q*) image and discrete cosine transform (DCT) energy image—were generated from these images. A final fused (FF) image combining *Q* and DCT of PP and GGO-PI images was then obtained. Five convolutional neural networks (CNNs) and five classic classification techniques, trained using FF and grayscale PP images, were tested. Our study is aimed at showing that a CNN model, with preprocessed images as input, has significant advantages over grayscale images. Previous work in this field primarily focused on grayscale images, which presented some limitations. This paper demonstrates how optimal results can be obtained by using the FF image rather than just the grayscale PP image. As a result, CNN models outperformed traditional artificial intelligence classification techniques. Of these, Vgg16Net performed best, delivering top-tier classification results for COVID-19 severity assessment, with a recall rate of 95.38%, precision of 96%, accuracy of 95.84%, and area under the receiver operating characteristic (AUROC) curve of 0.9585; in addition, the Vgg16Net delivers the lowest false negative (FN) results. The dataset, comprising 44 COVID-19 patients, was split equally, with half used for training and half for testing.

## 1. Introduction

The severe acute respiratory syndrome coronavirus 2 (SARS-CoV-2) outbreak, responsible for coronavirus disease 2019 (COVID-19), began in Wuhan in December 2019 and rapidly spread worldwide. On March 11, 2020, the World Health Organization classified it as a pandemic [1]. As of 2023, the ongoing emergence of new variants has resulted in 657,977,736 confirmed cases and 6,681,433 deaths [2]. Over the week from December 26, 2022, to January 1, 2023, there were more than 3 million new cases and 10,000 deaths reported [2].

**Figure 1:**
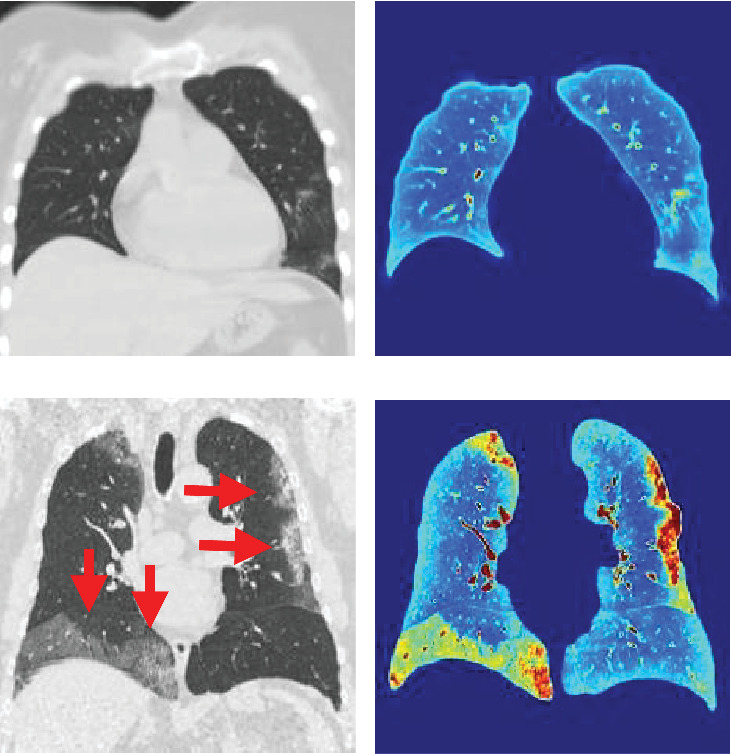
Computed tomography of the chest of a patient with nonserious COVID-19. (a) Without processing. (b) With processing. CT scan of a severe COVID-19 patient. (c) Without processing. (d) With processing.

In 2020, reverse transcription–polymerase chain reaction (RT-PCR) became the primary method for diagnosing COVID-19. It provides a reliable positive result [3]. Computed tomography (CT) emerged as an essential tool for identifying lung damage. It is advantageous in assessing the extent of lesions in infected patients [4]. Typically, critically hospitalized COVID-19 patients exhibit symptoms such as ground-glass opacity (GGO), consolidation, and peripheral infiltrates (PIs) accompanied by pleural effusion [5].  Figure 1(c) demonstrates these symptoms through CT images.

Chest CT scans allow radiologists and doctors to quickly identify coronavirus infection by recognizing visual markers and evaluating the scope of lung lesions [4]. This vital data can aid in creating an artificial intelligence (AI) application to supplement medical tools. Consequently, there is significant impetus to improve CT imaging using advanced image processing techniques, with the goal of increasing the precision of severity detection utilizing diverse AI methods.

Recent studies [4, 6–21] have reinforced the value of using CT evaluation to enhance patient treatment efficacy. Generally, these studies classify COVID-19 severity using various AI techniques and CT or X-ray images. They often apply these images directly, with little to no image preprocessing. In light of this, our study seeks to introduce a more precise method for gauging COVID-19 severity using CT scans. To improve the analysis of CT images and accurately determine COVID-19 severity, we use image processing techniques and deep learning. We focus on identifying the unique characteristics of ground-glass opacity and pulmonary infiltrates (GGO-PIs) (Figure 1(d)). These serve as key indicators in assessing COVID-19 severity within CT images. We specifically apply saliency (*Q*) and the discrete cosine transform (DCT) energy to the regions of pulmonary parenchyma (PP) and GGO-PI. This effectively highlights the relevant data in the affliction zones within the CT images. As we will demonstrate, our method has an added advantage over most current works in terms of applying the final fused (FF) color images to feed the convolutional neural networks (CNNs).

## 2. Related Works and Background

Numerous studies have been conducted since the start of the pandemic to examine the use of AI in analyzing CT scans of COVID-19 patients [5, 22–24]. Current research continues this trend with a concentration on CT scan data interpretation, which includes the detection and classification of COVID-19 as well as augmentation and analysis using image processing techniques [25].

For example, Alzahrani, Bhuiyan, and Akhter[4] put forward a method to enhance fuzzy CT scans by using fuzzy histogram equalization. This technique improved image contrast with the help of a normalized fuzzy image histogram. Kuanr et al. constructed a system that employed a ResNet CNN and image similarity to extract features from chest X-ray images for treating COVID-19 [6]. Additionally, Jabbar et al. suggested a modified unsharp masking technique to augment CT scans of COVID-19 patients, focusing on edges and discontinuities to boost contrast [7].

Prabha et al. developed a hybrid method, fusing swarm intelligence and fuzzy discrete particle swarm optimization (DPSO) algorithms, for analyzing COVID-19 infections through CT scans [8]. In a similar vein, Shabani et al. proposed a segmentation technique for COVID-19 CT images. Their method autogenerates a 3D pseudomask via a self-supervised model and uses a generative adversarial network (GAN) with U-Net as a multiobjective segmentation model to forecast lesions [9].

Perumal, Narayanan, and Rajasekar [5] suggested a hybrid learning model for categorizing CT images as COVID-19, typical pneumonia, or normal. The model blends machine-learning and deep-learning methods. Sukanya and Kamalanand evaluated four deep-learning techniques (ResNet101, ResNet50, ResNet18, and SqueezeNet) to classify and stage varied COVID-19 lung CT images [10]. Similarly, Abdulkareem et al. [11] created a COVID-19 diagnostic system that utilizes a combination of a CNN, a stacked autoencoder, and a deep neural network.

Faragallah, El-Hoseny, and El-Sayed presented an effective simple linear iterative clustering (SLIC) segmentation algorithm, which is based on the modified central force optimization (MCFO), to detect positive COVID-19 cases from chest CT images [12]. Nair et al. used a different approach, applying the K-means method for preprocessing CT images and the watershed method for segmenting the affected area. They then used the VGG-16 model for feature extraction, followed by a support vector machine (SVM) classifier to categorize COVID-19 pneumonia as mild, moderate, or severe [13].

Dutta et al. developed the efficient deep-learning-based fusion model with swarm intelligence (EDLFM-SI) a deep-learning model that uses swarm intelligence to identify SARS-CoV-2 from CT scans [14]. Guhan et al. combined a hybrid watershed and fuzzy c-means algorithms for lung CT image segmentation and then extracted texture features with the gray-level co-occurrence matrix (GLCM) algorithm. They used the Naïve Bayes classifier to sort these images into COVID and non-COVID categories [15]. Meanwhile, Gupta and Bajaj utilized the deep-learning model DarkNet19 to detect and classify COVID-19 in CT images [16]. Nur, Khan, and Nasir proposed a segmentation method that combined fuzzy c-means and fast fuzzy c-means hybrid, followed by a fusion technique. This technique extracted features using both the C transform and VGG19 CNN [17].

Chen et al. presented a method for COVID-19 infection segmentation, incorporating a unique data augmentation module based on the Fourier transform to manage intensity differences in CT images. Their approach included a teacher–student network designed for learning rotation-invariant features for segmentation [18]. Likewise, Suganya and Kalpana used the mask regional-CNN to categorize CT images as COVID-19 or non-COVID-19 [19]. Aswathy, Hareendran, and Vinod Chandra applied a ResNet-50 transfer learning network to detect COVID-19 in CT images and evaluated patient severity using pretrained ResNet-50 and DenseNet-201 networks for feature extraction, training a back-propagation neural network [20]. Similarly, Celik introduced a deep-learning-based model (CovidDWNet+GB) for COVID-19 detection using CT and X-ray images [21].

Our proposed methodology is aimed at improving upon previous developments in image processing and classification. We have developed a system that accentuates pertinent characteristics of GGO-PIs present in CT scans of COVID-19 patients. The system works in sync with a CNN to classify these scans into non-serious or severe categories. This was achieved by testing five distinct CNN models to determine the most suitable model. The system effectively converts CT scan results into insightful diagnostic images for medical professionals.

Additionally, the single-minded use of a CNN on unprocessed grayscale PP images is insufficient when compared to our method. Our approach emphasizes the fusion of *Q* and DCT with the CNN, which targets the GGO-PI features and enhances the diagnostic efficacy of our system. We also conducted parallel experiments using five traditional AI classification techniques on FF and grayscale PP images to underscore the advantages of employing CNNs.

## 3. Methods

### 3.1. Oversegmentation of the Image Identifying the PP

**Figure 2:**
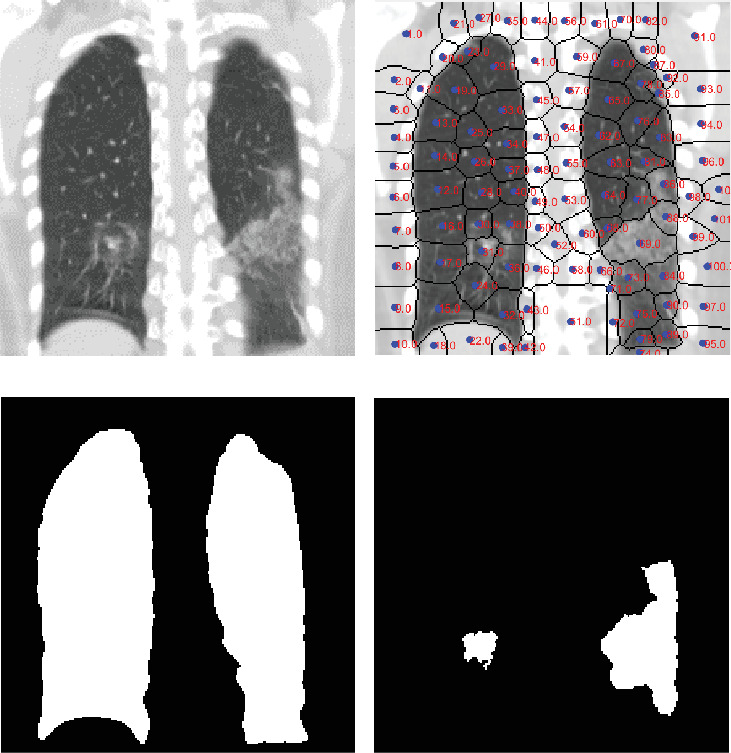
(a) Computed tomography of the chest of a patient with serious COVID-19. (b) Superpixel clusters for feature extraction. (c) Segmentation and identification of lung PP. (d) Regrouping and identification of the GGO-PI regions.

Drawing from the studies in [26], we introduce an automated segmentation technique for identifying the areas of the PP and consolidating the GGO-PIs within the CT images (Figure 2(a)). We first apply the mean shift algorithm to oversegment areas consisting of similar-intensity regions. We then execute a Superpixels algorithm that takes into account the image's intensity and spatial features (Figure 2(b)). Features such as position, grey intensity, second-order texture, and spatial context coherence are extracted from each cluster to segment the PP (Figure 2(c)). This segmentation is performed using a tree random forest (TRF). For both PIs and GGOs, known for their high gradient, we meld the watershed method with TRF, applying it to clusters within the PP area to consolidate the GGO-PI regions (Figure 2(d)).

### 3.2. Salience Fusion of GGO-PIs

**Figure 3:**
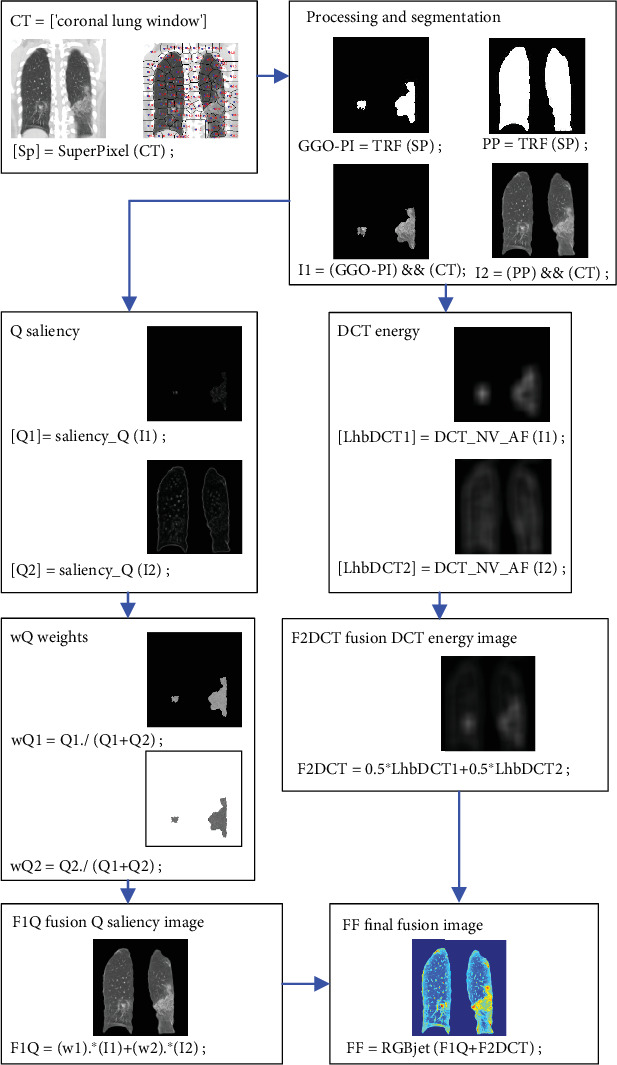
Block diagram of the implemented code.

**Figure 4:**
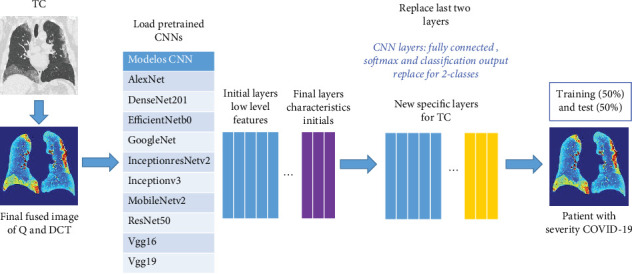
Overall method description for COVID-19 CT image severity classification.

Upon initially identifying the PP area (Figure 2(c)) and selecting a candidate region with severe GGO-PI (Figure 2(d)), we use *Q* and DCT to heighten the visibility of severity characteristics (Figure 3). By using *Q* and DCT images, we discern spatial frequency in varying image regions specifically. Then, we apply imaging fusion to synergistically combine information from the *Q* and DCT images. The resulting FF fused image is used in five CNN models [27] (Figure 4) to enhance patient classification results.

*Q* is an image that emphasizes the most relevant regions for deep-learning models. Its purpose is to indicate the importance of specific pixels.

The DCT expresses an image as a combination of sinusoids of different magnitudes and frequencies captured in two-dimensional 8 × 8 matrices. Most of the image's significant visual data is concentrated in the frequencies of the horizontal coefficients, which increase from left to right, and vertical coefficients, growing from top to bottom. The highest energy is found on the left of the high DCT.

Image fusion is a process that combines relevant, complementary, and redundant visual information from multiple sources to form a single composite image [28–30]. This procedure begins with the sharpening of the GGO-PI to enhance the target information in Image I1. The suggested method includes three steps for fusion: analysis and decomposition of the *Q* [31] and the DCT [32] of the CTs; fusion of the *Q* image and the DCT energy image; and the reassembly, synthesis, or fusion of the *Q* identified image and DCT energy image from the CT image in RGBjet format. This is subsequently inputted into the CNN. Figure 3 illustrates the proposed methodology with a block diagram, while Figure 4 displays the results of image processing from various CNN models.

In the stage of decomposition and analysis, the *Q* produced a detailed image, while the DCT was used to procure the energy image. These derived *Q* details and DCT energy were combined using several fundamental merging rules. The final product, a merged image known as FF, was reconstructed from the ultimate detail and energy images. Afterward, the RGBjet transform was implemented on the merged image in order to feed a CNN.

The initial images, PP and GGO-PI, are represented as I1 and I2, respectively. The first step in our method involves deriving the structure of saliency detail images, *Q*1 and *Q*2, from *I*1 to *I*2. To do this, we first calculate the local image structure using the local gradient covariance *C* and then generate the *Q* image measure.

(1) C=∑Ix2w3x3∑IxIyw3x3∑IxIyw3x3∑Ix2w3x3=Vs12  s12VT

(2) $$\begin{array}{c} Q=\sqrt{{\left(s_{1}+s_{2}\right)}^{2}+\alpha {\left(s_{1}+s_{2}\right)}^{2}} \end{array}$$

where *α* = 0.5 for controlling the shape of the contours. The covariance *C* of the local gradient is related to the structure of the image. *I*_*x*_(*w*) and *I*_*y*_(*w*) denote the gradients *x* and *y* over a local window *w*_[3 × 3]_. We decompose *C* through *s*_1_ and *s*_2_ (square roots of the eigenvalues) because high values of *s*_1_ and *s*_2_ indicate that the structure is sharp in two orthogonal directions.

From the salience *Q*1 and *Q*2, we obtain the weights of the images. These weights are calculated in different ways. The weight of GGO-PI (I1) is obtained by *w*1 = *Q*1/(*Q*1 + *Q*2), and the weight of PP (I2) is obtained by *w*2 = *Q*2/(*Q*1 + *Q*2). With each input image (I1 and I2), we can obtain the final salience detail (F1*Q*), from which we can merge the images. We calculate *F*1 as F1*Q* = *w*1∗*I*1 + *w*2∗*I*2. Figure 4 presents the steps and resulting images.

The second step involves calculating the DCT energy for the images, denoted as left high discrete cosine transform 1 (LhDCT1) and left high discrete cosine transform 2 (LhDCT2), respectively. We apply the DCT to the original PP and GGO-PI images (I1 and I2) to accomplish this. For each 8 × 8 DCT block (as shown in Equation (3)) of the I1 and I2 images, we compute the sum of the absolute values of half of the coefficients, namely AC coefficient (as shown in Equation (4)), AC_2_ to AC_32_ (the lowest-frequency coefficients). This computation is performed in a zigzag order from AC_2_ to AC_32_, which is sensitive to contours and less susceptible to noise (as depicted in the top left high part of the DCT in Equation (4)). Subsequently, a 3 × 3 median filter is applied to the reduced 8 × 8 image of accumulated AC coefficient values to mitigate the noise impact, resulting in the LhDCT1 and LhDCT2 energy images.

(3) $$\begin{array}{c} {\text{DCT}}_{\text{pq}}=\alpha_{p}\alpha_{q}\overset{M-1}{\underset{m=0}{\sum }}\overset{N-1}{\underset{n=0}{\sum }}A_{mn}\cos\left[\frac{\pi \left(2m+1\right)p}{2M}\right]\cos\left[\frac{\pi \left(2n+1\right)q}{2N}\right] \end{array}$$

(4) $$\begin{array}{c} {\text{DCT}}_{\text{pq}}=\left[\begin{array}{cccccccc} {\text{DC}}_{1} & {AC}_{2} & {AC}_{6} & {AC}_{7} & {AC}_{15} & {AC}_{16} & {AC}_{28} & {AC}_{29}\\ {AC}_{3} & {AC}_{5} & {AC}_{8} & {AC}_{14} & {AC}_{14} & {AC}_{27} & {AC}_{30} & {\text{AC}}_{43}\\ {AC}_{4} & {AC}_{9} & {AC}_{13} & {AC}_{18} & {AC}_{26} & {AC}_{31} & {\text{AC}}_{42} & {\text{AC}}_{44}\\ {AC}_{10} & {AC}_{12} & {AC}_{19} & {AC}_{25} & {AC}_{32} & {\text{AC}}_{41} & {\text{AC}}_{45} & {\text{AC}}_{54}\\ {AC}_{11} & {AC}_{20} & {AC}_{24} & {\text{AC}}_{33} & {\text{AC}}_{40} & {\text{AC}}_{46} & {\text{AC}}_{53} & {\text{AC}}_{55}\\ {AC}_{21} & {AC}_{23} & {\text{AC}}_{34} & {\text{AC}}_{39} & {\text{AC}}_{47} & {\text{AC}}_{52} & {\text{AC}}_{56} & {\text{AC}}_{61}\\ {AC}_{22} & {\text{AC}}_{35} & {\text{AC}}_{38} & {\text{AC}}_{48} & {\text{AC}}_{51} & {\text{AC}}_{57} & {\text{AC}}_{60} & {\text{AC}}_{62}\\ {\text{AC}}_{36} & {\text{AC}}_{37} & {\text{AC}}_{49} & {\text{AC}}_{50} & {\text{AC}}_{58} & {\text{AC}}_{59} & {\text{AC}}_{63} & {\text{AC}}_{64} \end{array}\right] \end{array}$$

The images LhDCT1 and LhDCT2 are crucial for the computation of the fusion DCT energy image (F2DCT) fusion Q saliency image (F1Q) which is used in the ultimate fusion of FF images. By averaging the LhDCT1 and LhDCT2 images, the F2DCT is generated. Additional information and the corresponding images are provided in Figure 3.

We can merge the *Q* detail image and DCT energy image by simply adding the fusion *Q* saliency image (F1*Q*) to the last base image (F2DCT). The result is a new image that amalgamates the sharpness of GGO-PI and PP (Images I1 and I2). This combined image is designated as FF. The image transformation is concluded by utilizing the RGBjet color scheme (Figure 3).

The final fusion method yields a fused image (FF in RGBjet), which is used as input for the CNN models. These models are trained on CT images that have been processed through connected region merging, adhering to our criteria and focusing on relevant and intensified GGO-PI regions of interest. Our approach is aimed at maximizing recall to reduce false negatives (FNs) and increase precision to identify or eliminate false candidates using deep-learning techniques. This is a key goal in medical imaging. We have designed specific values for predefined constraints, ensuring flexibility to avoid losing true positives (TPs).

### 3.3. COVID-19 Patient Severity Classification

#### 3.3.1. CNN Models

We introduce a proficient preprocessing technique to emphasize the regions of interest for GGO-PI in CT scans. The highlighted areas are subsequently utilized as input for CNN models, run on MATLAB, with the final layer modified through a transfer learning method (Figure 4).

Transfer learning hinges on a correlation between the pretrained model (such as AlexNet, GoogleNet, Res50Net, VGG16Net, and VGG19Net) and the desired task domain, which in this case is computer vision. The process begins by loading a pre-existing model and downloading its network weights. The final output layer is then removed, and new trainable final output layers are added to adapt it to the number of classes to be classified. Various preloaded models typically generate about 1000 output classes; the model is subsequently retrained with this new output layer to suit a two-class system.

LeCun et al. [27] first proposed a CNN model to recognize characters. A CNN model is a distinct type of neural network that uses convolution operations for processing. It merges automatic feature extraction with the classification abilities of a multilayer perceptron.

This study compares five distinct CNN models: AlexNet, GoogleNet, ResNet50, Vgg16, and Vgg19 (Figure 4). The use of these models, which apply convolution operations to input images to identify key features, is based on previous research. A multilayer perceptron classifier then uses these features. In our approach, we modified the final layers of these CNN models to improve classification accuracy, recall, and precision.

Our method leverages preprocessed images as input, proving more effective and resilient than traditional methods that solely use grayscale images. Additionally, using preprocessed 3D images for network feeding presents notable advantages, setting our work apart from others that rely only on grayscale images.

This study primarily contributes by generating input images and extracting relevant GGO-PI information to train the CNN. It also creates a representative dataset for training and evaluates multiple classification strategies (AlexNet, GoogleNet, ResNet50, Vgg16, and Vgg19) for solving the classification problem.

**Figure 5:**
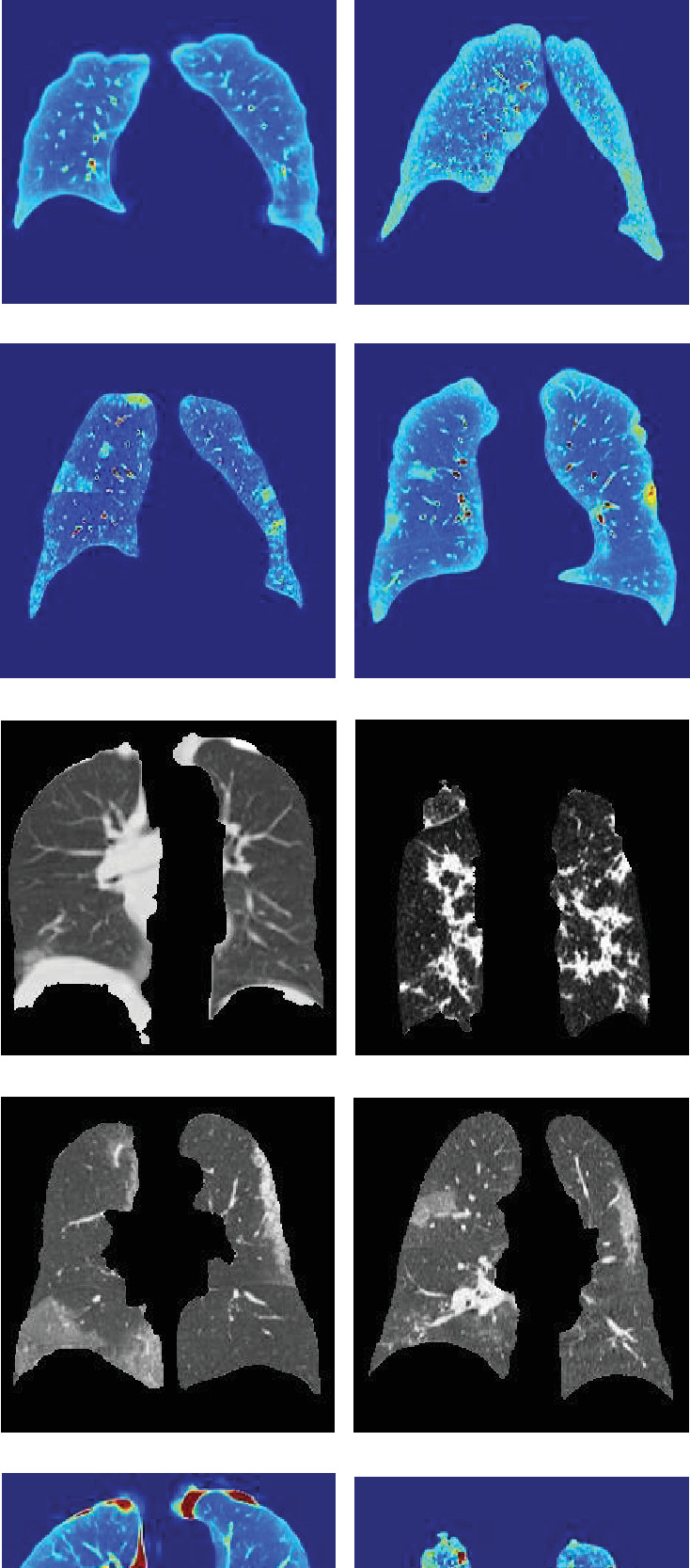
Fusion image results of *Q* and DCT. (a–d) Grayscale PP without severity image results without processing. (e–h) FF image result in processing. (i–l) Grayscale PP with severity image results without processing. (m–p) FF image result in processing.

Transfer learning employs a pretrained network on a large, easily available dataset to form a representation for learning a new task, eliminating the need to start training from scratch. In our study, we modified these pretrained networks to distinguish between CT images. We accomplished this by replacing all layers after the last convolution block, such as the fully connected, softmax, and classification output layers (Figure 4), with new layers of suitable dimensions. The main goal was to categorize CT images from COVID patients by severity, distinguishing between two distinct image classes: severe (Figures 5(i), 5(j), 5(k), and 5(l)) and nonsevere (Figures 5(a), 5(b), 5(c), and 5(d)). For example, severe CT images are displayed in Figures 5(i), 5(j), 5(k), and 5(l) (raw) and Figures 5(m), 5(n), 5(o), and 5(p) (FF processed), while nonsevere images are shown in Figures 5(a), 5(b), 5(c), and 5(d) (raw) and Figures 5(e), 5(f), 5(g), and 5(h) (FF processed). Next, we fine-tuned the adapted networks using custom training instances for this new task.

Table 1 presents the results of deploying the five CNN models. It highlights the advantages of utilizing the processed CT database (FF images, shown in Figures 5(e), 5(f), 5(g), 5(h), 5(m), 5(n), 5(o), and 5(p)) over the unprocessed CT database (grayscale PP images, depicted in Figures 5(a), 5(b), 5(c), 5(d), 5(m), 5(n), 5(o), and 5(p)).

#### 3.3.2. Conventional AI Techniques

We conducted classification experiments on the same database using five widely used AI classification techniques: TRF, tree logistic model tree (LMT), tree J48, multilayer perceptron, and Bayes net. These tests were run using Weka (Waikato Environment for Knowledge Analysis). These traditional methods first characterized the PP zone (Figure 2(c)). This involved a grayscale texture features vector for grayscale PP images (Figures 5(a), 5(b), 5(c), 5(d), 5(m), 5(n), 5(o), and 5(p)) and an RGB vector for the FF image (Figures 5(e), 5(f), 5(g), 5(h), 5(m), 5(n), 5(o), and 5(p)).

One approach for classifying CT (computed tomographic images) from a patient with nonserious or severe COVID-19 involves comparing first and second-order texture levels. This comparison is conducted across the three layers RGB∗ in the case of the FF image and using grayscale in the context of grayscale PP images. First-order features are computed individually based on pixels *p*(*i*, *j*). These include mean *μ* (5), median *m* (6), variance *σ*^2^ (7), standard deviation *σ* (8), and entropy *S* (9). Second-order characteristics are extracted from the Haralick co-occurrence matrix *c*(*i*, *j*). These characteristics encompass the contrast descriptor CM (10), correlation *r* (11), energy *e* (12), and local homogeneity HL (13).

(5) $$\begin{array}{c} \mu =\frac{1}{\text{ij}}\underset{i,j}{\sum }p\left(i,j\right) \end{array}$$

(6) $$\begin{array}{c} m=L+I\left(\frac{\left(N/2\right)-F}{f}\right) \end{array}$$

(7) $$\begin{array}{c} \sigma^{2}=\frac{1}{\text{ij}}\underset{i,j}{\sum }\left(p\left(i,j\right)-\mu \right) \end{array}$$

(8) $$\begin{array}{c} \sigma =\sqrt{\frac{1}{\text{ij}}\underset{i,j}{\sum }\left(p\left(i,j\right)-\mu \right)} \end{array}$$

(9) $$\begin{array}{c} S=-\underset{i,j}{\sum }p\left(i,j\right)-\log p\left(i,j\right) \end{array}$$

(10) $$\begin{array}{c} \text{CM}=\underset{i,j}{\sum }\left|i-j\right|c\left(i,j\right) \end{array}$$

(11) $$\begin{array}{c} r=\underset{i,j}{\sum }\frac{\left(i-\mu_{\text{ci}}\right)\left(j-\mu_{\text{cj}}\right)c\left(i,j\right)}{\sigma_{\text{ci}}\sigma_{\text{cj}}} \end{array}$$

(12) $$\begin{array}{c} e=\underset{i,j}{\sum }c{\left(i,j\right)}^{2} \end{array}$$

(13) $$\begin{array}{c} \text{HL}=\underset{i,j}{\sum }\frac{c\left(i,j\right)}{1+\left|i-j\right|} \end{array}$$

The first-order texture gave 15 features (*μ*_*R*_, *μ*_*G*_, *μ*_*B*_, *m*_*R*_, *m*_*G*_, *m*_*B*_, *σ*^2^_*R*_, *σ*^2^_*G*_, *σ*^2^_*B*_, *σ*_*R*_, *σ*_*G*_, *σ*_*B*_, *S*_*R*_, *S*_*G*_, and *S*_*B*_), and the second-order texture gave 12 features (CM_*R*_, CM_*G*_, CM_*B*_, *r*_*R*_, *r*_*G*_, *r*_*B*_, *e*_*R*_, *e*_*G*_, *e*_*B*_, HL_*R*_, HL_*G*_, and HL_*B*_), which were classified via the FF image RGB∗ levels to give 27 features. Thus, for the grayscale PP image, there are nine texture features (*μ*_*G*_, *m*_*G*_, *σ*^2^_*G*_, *σ*_*G*_, *S*_*G*_, CM_*G*_, *r*_*G*_, *e*_*G*_, and HL_*G*_). In order to classify these features, we explored five conventional AI classification techniques through Weka. The experimental results obtained (Table 2) are compared with the results of the CNNs.

## 4. Experimental Results

### 4.1. Dataset Description

To determine the ground truth, the medical team carefully examined all CT images from the COVID-19 patient database, distinguishing between images showing severe lung damage and those without. The grouping of these images relied on diagnostic evaluation and visual estimation of the observed damage.

We used a dataset of 2308 CT images from 44 COVID-19 patients. These images were categorized according to the severity seen in each case; 1168 CT images showed severe damage, and 1140 were classified as moderate. All the patients had tested positive for COVID-19 via RT-PCR testing.

Our study primarily focused on spotting large GGO lesions in the peripheral and posterior lung regions across all CT images. These lesions are considered a diagnostic sign of COVID-19 pneumonia in the appropriate clinical context.

The dataset images were in grayscale JPEG format and resized to fit the dimensions needed by the respective CNN model or FF processing. Samples from the dataset are displayed in Figures 1 and 5.

We split the dataset equally for training and testing, assigning 50% each. We used MATLAB for the CNN models, and Weka for traditional AI classification approaches. Class 1 indicated severe damage, and Class 2 represented nonsevere cases. The test phase outcomes are shown in Tables 1 and 2, respectively.

### 4.2. Quality Indicators of Computed Thermographic Image Classification of COVID-19 Severity

We present a confusion matrix summarizing the correct and incorrect predictions of our CNN models. Table 1 provides a comprehensive summary of all our quantitative results. When compared with traditional AI classification techniques (outlined in Table 2), our CNN models showcase superior performance.

First, we defined the positive detection *P* as the number of CT images with severity in the dataset: (i) the number of TPs is TP; (ii) the number of false positives is FP; (iii) the number of FNs is FN. Using these metrics, the classification performance is obtained in terms of sensitivity, recall, or true positive rate (TPR) (TPR = TP/P); precision or positive predictive value (PPV) (PPV = TP/TP + FP); false discovery rate (FDR = FP/FP + TP); and *F*1 score and overlap or harmonic mean (HM) of TPR and PPV (F1 = 2^∗^TP/2^∗^TP + FP + FN and HM = 2^∗^TPR^∗^PPV/TPR + PPV, respectively).

Second, we defined *N* as the number of CT images without severity resulting from applying the proposed method to the complete dataset: (iv) TN is the number of true negatives after classification. Adding this metric gives the classification performance in terms of specificity or true negative rate (SPC = TN/*N*); negative predictive value (NPV = TN/TN + FN); accuracy (ACC = TP + TN/TP + FP + TN + FN); and fall-out or false positive rate (FPR = FP/*N*).

Tables 1 and 2 also include the area under the receiver operating characteristic (AUROC) metric to quantify the classifier's performance at all possible operating points.

This study examined five CNN models, finding Vgg16Net to yield the most competitive and reliable results in the practical testing experiment (Table 1); the principal goal was to classify the severity of lung damage, and Vgg16Net presents the lowest FN rate. The dataset was split, with 50% allocated for training and the remainder for testing. These results evidence the proficiency of Vgg16Net in distinguishing COVID-19 CT images in comparison to other CNN models and traditional AI techniques (Tables 1 and 2).

The key contributions of this study include (i) identifying salient features from lung CT scans of COVID-19 patients, notably the GGO-PI; (ii) enhancing images by fusing pulmonary parenchyma (PP) and GGO-PI images using *Q* images and DCT energy images (Figure 5); (iii) using 2308 CT images to assess the severity of COVID-19; and (iv) offering the outcomes of the most effective CNN model for classifying CT images. This aids in tracking disease progression and interpreting the severity of COVID-19.

The results underline the benefits of employing full-color images, rather than grayscale, across both CNN models and traditional AI methods. Additionally, CNN models demonstrate advantages compared to conventional AI techniques.

## 5. Conclusions

Evaluating the severity of pulmonary damage in COVID-19 patients' CT scans is crucial for diagnosis. Our study presents a Vgg16Net CNN–based approach for classifying these images, which exhibits outstanding, consistent results: 95.38% recall, 96% precision, 95.84% accuracy, and an AUROC curve of 0.9585; and the lowest FN rate. For this methodology, the Vgg16Net model was applied to half of the dataset (22 COVID-19 patients), with the other half used for testing (a separate group of 22 COVID-19 patients). We employed an innovative fusion technique that enhanced the CT image analysis. Given the lack of a fully standardized comparison framework, this evaluation reasonably affirms the novelty and quality of our results. Utilizing the fusion method for image preprocessing led to top-class accuracy with the CNN, making the Vgg16Net model the most effective for image classification. Further research could delve into the potential insights these CNN models provide in gauging the progression of disease severity in CT scans. This study argues convincingly for the integration of full-color (FF) processed images with CNN models.

## Figures and Tables

**Table 1:** Confusion matrix of testing datasets and results (percent) of quantitative classification experiment of CT with CNN models.

**CNN model confusion matrix**				**TPR**	**PPV**	**FDR**	**F1/HM**	**SPC**	**NPV**	**FDR**	**ACC**	**AUROC**
With processing (FF images)												
AlexNet				96.23%	94%	6.02%	95.09%	94%	96.04%	6.32%	94.97%	94.96%
TP	562	FN	22									
FP	36	TN	534									
GoogleNet				92.98%	94%	5.73%	93.62%	94%	92.91%	5.79%	93.59%	93.59%
TP	543	FN	41									
FP	33	TN	537									
res50Net				90.58%	98%	2.04%	94.13%	98%	91.04%	1.93%	94.28%	94.33%
TP	529	FN	55									
FP	11	TN	559									
VGG16Net				95.38%	96%	3.63%	95.87%	96%	95.31%	3.68%	95.84%	95.85%
TP	557	FN	27									
FP	21	TN	549									
VGG19Net				98.46%	89%	11.2%	93.34%	87%	98.22%	12.81%	92.89%	93%
TP	575	FN	9									
FP	73	TN	497									
Without processing (grayscale PP images)												
AlexNet				92.64%	95%	4.92%	93.84%	95%	92.65%	4.91%	93.85%	93.86%
TP	541	FN	43									
FP	28	TN	542									
GoogleNet				93.49%	91%	9.00%	92.23%	91%	93.14%	9.47%	92.03%	92%
TP	546	FN	38									
FP	54	TN	516									
res50Net				90.92%	91%	9.08%	90.92%	91%	90.70%	9.30%	90.81%	90.81%
TP	531	FN	53									
FP	53	TN	517									
VGG16Net				98.63%	88%	12.20%	92.90%	86%	98.39%	14.04%	92.37%	92%
TP	576	FN	8									
FP	80	TN	490									
VGG19Net				78.08%	98%	1.51%	87.11%	99%	81.48%	1.23%	88.30%	88.43%
TP	456	FN	128									
FP	7	TN	563									

Abbreviations: ACC, accuracy; AUROC, area under the receiver operating characteristic; CNN, convolutional neuronal network; F1, F1 score; FDR, false discovery rate; FPR, fall-out or false positive rate; HM, harmonic mean; NPV, negative predictive value; PPV, precision or positive predictive value; SPC, specificity or true negative rate; TPR, sensitivity, recall, or true positive rate.

**Table 2:** Confusion matrix of testing datasets and results (percent) of quantitative classification experiment of CT with conventional AI classification models.

**CNN model confusion matrix**				**TPR**	**PPV**	**FDR**	**F1/HM**	**SPC**	**NPV**	**FDR**	**ACC**	**AUROC**
With processing (FF images)												
Tree random forest				84.35%	90%	10.31%	86.94%	90%	84.69%	10.07%	87.09%	95.10%
TP	496	FN	92									
FP	57	TN	509									
Tree LMT				84.01%	92%	8.18%	87.74%	92%	84.74%	7.77%	88.04%	95.80%
TP	494	FN	94									
FP	44	TN	522									
Tree J48				86.73%	89%	11.30%	87.70%	89%	86.53%	11.48%	87.61%	87.40%
TP	510	FN	78									
FP	65	TN	501									
Multilayer perceptron				85.03%	89%	10.87%	87.03%	89%	85.16%	10.78%	87.09%	94.70%
TP	500	FN	88									
FP	61	TN	505									
Bayes net				82.65%	80%	19.54%	81.54%	79%	81.45%	20.85%	80.94%	90%
TP	486	FN	102									
FP	118	TN	448									
Without processing (grayscale PP images)												
Tree random forest				85.71%	87%	13.10%	86.30%	87%	85.37%	13.43%	86.14%	94.30%
TP	504	FN	84									
FP	76	TN	490									
Tree LMT				83.33%	89%	10.91%	86.12%	89%	83.77%	10.60%	86.31%	94%
TP	490	FN	98									
FP	60	TN	506									
Tree J48				87.41%	84%	15.60%	85.88%	83%	86.42%	16.78%	85.36%	88.80%
TP	514	FN	74									
FP	95	TN	471									
Multilayer perceptron				83.50%	90%	9.74%	86.75%	91%	84.10%	9.36%	87.00%	94%
TP	491	FN	97									
FP	53	TN	513									
Bayes net				80.61%	81%	19.25%	80.68%	80%	79.89%	19.96%	80.33%	89%
TP	474	FN	114									
FP	113	TN	453									

Abbreviations: ACC, accuracy; AUROC, area under the receiver operating characteristic; CNN, convolutional neuronal network; F1, F1 score; FDR, false discovery rate; FPR, fall-out or false positive rate; HM, harmonic mean; NPV, negative predictive value; PPV, precision or positive predictive value; SPC, specificity or true negative rate; TPR, sensitivity, recall, or true positive rate.

## Data Availability

The data used to support the findings of this study are available from the corresponding author upon request. Computed tomography images (data) are provided for download in JPG. The authors provide data for the scientific community interested in conducting research on the severity classification of the lung parenchyma of patients with COVID-19. This data can be used to compare various image analysis techniques, generating solid conclusions, which is the subject of our work. The data contains all quantitative classification results for download (which can be requested from the corresponding author): aOrigi-nal_image_GGO-PI_PP contains the entire original computed tomography image database, as well as the ground truth result data for every PP and GGO-PI (as jpg images); bFUSION_Q-DCT_CODE presents the original code for saliency detail layers, DCT energy maps, and FF image of *Q* and DCT to feed the CNN (saliency_Q, DCT_NV_AF, and FFCOVIDimages_to_CNN_2_0, respectably as Matlab.m); cPROCESSING_images_RESULTS is the entire original computed tomography image database and also contains the FF image results (as jpg images); inside the dMatlabCNN are the codes of the CNN models organized by folders that contain the resized images and classified by medical specialists (Classes 1–2, with or without severity); in addition, the results of the confusion matrix of each CNN model are presented (ResultadosMatrizConsusion-MATLAB.xlsx); inside the eWekaCLASSIC are the codes ClasicClass.m (for texture feature extraction) and COVIDDatasetFeatures1 and COVIDDatasetFeatures2 for Weka experiments (with processing and without processing) and the results of the confusion matrix of each conventional AI technique ResultadosMatrizConsusionWEKA.xlsx.
